# IL-6 as a driver of bone invasion in IFIT2-depleted oral squamous cell carcinoma

**DOI:** 10.1007/s00262-025-04234-6

**Published:** 2026-01-31

**Authors:** Kuo-Chu Lai, Chia-Hsun Hsieh, Chen-Hsuan Wang, Sheng-Yen Hsiao, Wan-Chen Kao, Chia-Chia Chao, Po-Chun Chen

**Affiliations:** 1 Department of Physiology and Pharmacology, College of Medicine, Chang Gung University, Taoyuan City, 33302 Taiwan; 2 Division of Hematology and Oncology, Chang Gung Memorial Hospital, Linkou, New Taipei City, 33305 Taiwan; 3 School of Life Science, National Taiwan Normal University, Taipei, 106 Taiwan; 4 Division of Hematology-Oncology, Department of Internal Medicine, Chi Mei Medical Center, Liouying, Tainan, 736 Taiwan; 5 Institute of Clinical Medicine, College of Medicine, National Cheng Kung University, Tainan, 701 Taiwan; 6 Department of Respiratory Therapy, Fu Jen Catholic University, New Taipei City, 242 Taiwan; 7 Translational Medicine Center, Shin-Kong Wu Ho-Su Memorial Hospital, Taipei City, 111 Taiwan; 8 Department of Medical Research, China Medical University Hospital, China Medical University, Taichung, 404 Taiwan

**Keywords:** IFIT2, OSCC, IL-6, Bone invasion, Osteoclasts

## Abstract

Oral squamous cell carcinoma (OSCC), the most prevalent oral cancer, often exhibits local bone invasion. Interferon-induced protein with tetratricopeptide repeats 2 (IFIT2) has been identified as a tumor suppressor in OSCC, though its role in bone invasion remains unclear. This study aimed to investigate the involvement of IL-6 as a driver of bone invasion in OSCC with depleted IFIT2 expression and to evaluate the clinical relevance of IFIT2 and IL-6. IFIT2 knockdown significantly increased IL-6 mRNA and secretion levels in OSCC cells, indicating IFIT2’s potential regulatory role in inflammation within the tumor microenvironment. Elevated IL-6 levels in IFIT2-depleted OSCC cells markedly enhanced osteoclast differentiation and bone resorption. Neutralizing IL-6 reversed these effects, confirming IL-6 as a key mediator of bone invasion in OSCC cells. These results indicate that IL-6 as driver of bone invasion in IFIT2-depleted OSCC cells. Clinically, serum IL-6 concentrations were significantly higher in patients with advanced-stage tumors (T4) compared to early stages (T1–3; *p* = 0.027), supporting IL-6’s role as a progression marker. Stage IV OSCC patients exhibited higher IL-6 levels compared to stages I–III, further substantiating IL-6’s prognostic significance in advanced OSCC (*p* = 0.043). Serum IL-6 positively correlated with pro-tumorigenic cytokines, including VEGF-α and G-CSF. Notably, a high IL-6/IFIT2 ratio was associated with reduced median survival among OSCC patients. These findings indicate that IFIT2 depletion enhances OSCC-induced bone invasion predominantly through IL-6-mediated osteoclast activation, establishing the IL-6/IFIT2 axis as a critical regulator of OSCC progression and metastasis.

**Supplementary Information:**

The online version contains supplementary material available at 10.1007/s00262-025-04234-6.

## Introduction

Oral squamous cell carcinoma (OSCC) is one of the most common cancers in the head and neck region, known for its aggressive nature and strong tendency for local invasion. One of the critical aspects of OSCC progression is bone invasion, which significantly affects prognosis and treatment outcomes [1]. The mechanism behind OSCC-associated bone invasion is complex, involving both direct tumor infiltration and an osteolytic process mediated by osteoclasts [2]. Osteoclasts, the primary cells responsible for bone resorption, play a key role in tumor-induced bone destruction. OSCC cells are known to release various pro-osteoclastogenic factors, including receptor activator of nuclear factor kappa-Β ligand (RANKL), interleukin-6 (IL-6), IL-11, and parathyroid hormone-related protein (PTHrP). These factors promote osteoclast differentiation and activation, leading to bone degradation and further facilitating tumor invasion [3]. In addition to IL-6, IL-11, and PTHrP, other tumor-secreted factors such as tumor necrosis factor (TNF)-α, IL-1, and macrophage chemotactic protein-1 (MCP-1) show elevated expression in OSCC [4–6]. Previous research has proved these secreted factors are involved in osteoclast formation [7, 8], suggesting their potential role in promoting osteolysis and subsequent bone invasion. By recruiting and activating osteoclast precursors, these molecules may further enhance local bone destruction in OSCC. The osteolysis in OSCC, in turn, releases growth factors stored in the bone matrix, which fuel further tumor proliferation and invasion. This reciprocal interaction between cancer cells and osteoclasts, often referred to as the “vicious cycle,” exacerbates both bone loss and tumor progression [9, 10]. Disrupting this cycle is therefore a critical therapeutic target for mitigating osteolytic damage and improving clinical outcomes in OSCC [2].

Cytokines play a multifaceted role in bone invasion by modulating osteoclast activity, promoting angiogenesis, and facilitating tumor progression [11].Cytokines can be classified into six groups: (1) L1 superfamily; (2) TNF superfamily; (3) IL-17 family; (4) IL-6 superfamily; (5) type I superfamily; and (6) type II superfamily [12]. Cytokines such as IL-6, TNF-α, and parathyroid hormone-related protein (PTHrP) are elevated in OSCC tumor cells. Additionally, conditioned media from these cells promote osteoclast differentiation and bone resorption in vitro [13, 14]. Therefore, targeted therapies aimed at disrupting these cytokine pathways hold promise for mitigating bone invasion and improving clinical outcomes in OSCC and other malignancies. Cytokines orchestrate immune responses and play a dual role in tumor biology, acting as both tumor promoters and suppressors depending on the context. Blocking VEGF-Flt-1 signaling may help inhibit bone invasion in OSCC, as VEGF produced by OSCC cells directly activates the Flt-1 pathway in pre-osteoclasts, promoting their migration to future bone-resorbing areas and differentiation into osteoclasts [15]. TGF-β and epidermal growth factor (EGF) are key regulators of epithelial–mesenchymal transition (EMT), a critical process in bone invasion by malignant epithelial cells. EMT enhances cancer cell migration, facilitating stromal invasion, intravasation, and subsequent dissemination [16]. The net effect of the inflammatory response is determined by the balance between pro-inflammatory and anti-inflammatory cytokines. Due to the simplicity and cheapness of accurately measuring cytokines, cytokine profiling is a valuable new tool that can complement other laboratory parameters used in the management of OSCC patients.

Interferon-induced protein with tetratricopeptide repeats 2 (IFIT2) has been reported to be an ISG protein and plays an antiviral role [17]. We are the first to demonstrate that IFIT2 expression was decreased in betel nut-exposed keratinocytes and further investigated the biological function of IFIT2 in OSCC. IFIT2 depletion resulted in EMT, chemoresistance, and enhanced in vivo angiogenesis and metastatic colonization [18–20]. Moreover, the expression of TNF-α was significantly upregulated in IFIT2-depleted HNC cells [20]. TNF-α is among the most important signaling elements influenced by IFIT2 expression, and its roles in the immune response have also been implied [21, 22]. Currently, our preliminary results demonstrate that metastatic IFIT2-depleted OSCC tumor can induce cachexia symptoms in NOD-SCID mice [23]. Our metastatic cancer-induced cachexia model will help identify molecular mediators that can effectively target HNC cachexia. Similar to our findings, IFIT2 downregulation is associated with tumor progression and poor survival in different types of cancer [24–26]. These results indicate that IFIT2 may play important role in tumor progression.

So far, few studies have demonstrated the involvement of IFIT2 in bone invasion by OSCC cells. This study aimed to investigate the changes in cytokine profiles following IFIT2 knockdown and their clinical relevance in OSCC patients. Knockdown of IFIT2 led to marked increases in pro-inflammatory cytokines such as IL-6 and TNF-α. These findings highlight the dual role of cytokines in OSCC progression and suggest IFIT2 as a potential regulator of inflammation and tumor suppression. Furthermore, neutralization of IL-6 effectively reversed osteoclastogenesis induced by IFIT2-deficient OSCC cells. Clinically, elevated serum IL-6 levels correlated with pro-tumorigenic cytokines such as VEGF-α and G-CSF, suggesting that IL-6 may act as a driver of bone resorption by promoting osteoclast differentiation. These findings suggest a regulatory role for IFIT2 in IL-6-related bone invasion in OSCC cells.

## Materials and methods

### Cell culture

Sh-control and stable sh-IFIT2#1 and sh-IFIT2#2 cells were generated in the human oral cancer cell line CAL27 as previously described [20]. Murine RAW 264.7 cells, a macrophage cell line derived from mice, were obtained from the Bioresource Collection and Research Center (BCRC) in Taiwan. They were cultured in Dulbecco’s Modified Eagle’s Medium (DMEM) supplemented with 10% fetal bovine serum (FBS), along with 100 U/mL penicillin and 100 μg/mL streptomycin.

### Preparation of conditioned medium (CM)

Protocol was carried out as previously described [27]. When the sh-control, sh-IFIT2#1, and sh-IFIT2#2 sublines reach a confluency > 80%, the growth medium was removed and then wash the cells twice with PBS. After incubating with DMEM medium without serum for 24 h, the medium was collected and centrifuged at 4500 rpm for 15 min. Aliquots of the filtered medium will be stored at -80℃.

### Cytokine profiling

HCYTMAG-60 K-PX41 (Millipore) was used to determine the circulating levels of human cytokines, respectively, of conditioned media from sublines or sera from OSCC patients. 100 μl per sample was carried out serum cytokine profiling by Hong Jing Co., LTD (Taipei, Taiwan).

### Osteoclast differentiation

Osteoclast differentiation assay was performed by culturing 750 cells/well of RAW 264.7 cells in a 48-well plate in Dulbecco’s Modified Eagle’s Medium supplemented with 10% FBS, 100U/ml penicillin, and 100 μg/ml streptomycin for 4 days. All cells were incubated with RANKL (50 ng/ml) and exposed to 10% CM from OSCCs in the presence with IL-6-neutralizing antibody or IgG control. The culture medium was replaced every 2 days, and osteoclast formation was counted after 4 days. Osteoclast differentiation was measured by determining the number of cells positively stained for tartrate-resistant acid phosphatase (TRAP; Acid Phosphatase Kit 387-A; Sigma–Aldrich) according to the manufacturer’s instructions. The total and average area of TRAP staining osteoclasts were quantified by ImageJ software (National Institutes of Health, USA).

### Actin ring staining for mature osteoclasts derived from RAW264.7 cells

To assess actin ring formation, osteoclasts were prepared as described in osteoclast differentiation. The osteoclasts were fixed with 4% paraformaldehyde for 15 min at room temperature. Actin rings were then visualized by staining with F-Actin Staining Kit (KA4116; Abnova; Taipei, Taiwan) for 30 min in the dark. Fluorescence images were captured using a Nikon ECLIPSE Ts2 microscope (Nikon; Tokyo, Japan) and analyzed with ImageJ software to quantify actin ring formation.

### Quantitative real-time PCR (Q-PCR)

Q-PCR was performed as previously described [20]. Total RNA was isolated using the TRI reagent (Molecular Research Center) and converted into cDNA using an oligo (dT) primer and the SuperScript III first-strand synthesis system (Life Technologies, Camarillo, CA, USA), Q-PCR was performed using a LightCycler 480 system (Roche Diagnostics, Pleasanton, CA, USA). The respective forward and reverse primers used for Q-PCR were 5'-ACAAGGCCATCCACCACTTTAT-3' and 5'-CCCAGCAATTCAGGTGTTAACA-3' for IFIT2, and 5'- GGAGTCCCTGCCACACTCA-3' and 5'- GCCCCTCCCCTCTTCAAG -3' for GAPDH.

### Western blotting analysis

Total cell lysates from various experimental groups were collected and separated using SDS-PAGE. The separated proteins were transferred to a PVDF membrane, which was then blocked using 4% BSA at room temperature for one hour. The membrane was further incubated for another hour at room temperature with a primary antibody diluted to a 1:1000 concentration against integrin β3 (ITGB3) (GTX02823; GeneTex; CA, USA), matrix metalloproteinase-9 (MMP-9) (GTX04573; GeneTex), cathepsin K (CTSK) (ab19027; Abcam; Cambridge, UK), and β-actin (A5441; Sigma; MA, USA). An HRP-conjugated anti-rabbit secondary antibody was applied at a 1:1000 dilution and incubated for one hour at room temperature, followed by three washes with TBST. Afterward, the membrane underwent three final washes in TBST before being developed using an enhanced chemiluminescence substrate. Protein bands were visualized using a CCD-based imaging system (MultiGel-21, TOP BIO CO., New Taipei City, Taiwan), and densitometric analysis was performed using ImageJ software.

### In vivo metastasis model

Animal care was approved by and followed the guidelines of the Institutional Animal Care and Utilization Committee of Tzu-Chi University. The male NOD/SCID mice were obtained from the animal center of Tzu-Chi University (Hualien, Taiwan). NOD/SCID mice were injected intravenously with 5 × 10^5^ sh-control or sh-IFIT2 cells. NOD/SCID mice without tumor injection act as control group. Eight weeks after tail vein injection, sh-IFIT2 cells were observed to form tumors in multiple anatomical sites, including the head, neck, thoracic cavity, peritoneal cavity, and retroperitoneal space. These cells also exhibited invasive behavior, infiltrating lymph nodes, bone, and muscle tissues. In contrast, sh-control cells predominantly developed tumors confined to the lungs [18]. At this time point, sera were collected via cardiac puncture for subsequent cytokine profiling.

### Micro-computed tomography (μCT) analysis

Following euthanasia, the spinal segments bearing tumor tissue were dissected and preserved in 4% paraformaldehyde for μCT evaluation. The spine tissues were subjected to scanning using a Skyscan 1276 system (Bruker, Kontich, Belgium). Raw projection data were processed and converted into cross-sectional images with a GPU-accelerated reconstruction tool (NRecon, Bruker). During this step, the grayscale was calibrated against the Hounsfield scale, with verified calcium standards serving as density references. Subsequently, the three-dimensional reconstructions were analyzed using CTAn software (Bruker) to quantify bone volume and determine the extent of osteolytic changes associated with tumor infiltration.

### Clinical sample recourse

47 OSCC sera were obtained and analyzed in accordance with the regulations and approval of the Chang Gung Memorial Hospital (IRB202100723B0C501) for cytokine profiling. To address expression correlation of IFIT2 and IL-6, an oral cavity cancer tissue array (ORC1021; Pantomics, Richmond, CA) containing 73 cases of oral cavity cancer, 18 cases of pharynx or hypopharynx cancer, and 11 cases of normal oral cavity tissues was used for immunohistochemically (IHC) analysis. All the clinical information associated with the array samples was delinked, resulting in the unavailability of survival data. Thus, the Cancer Genome Atlas (TCGA) database (http://ualcan.path.uab.edu/index.html) was used to analyze the diagnostic impact of IL-6 and IFIT2 in HNC, including OSCC.

### IHC analysis

The paraffin-embedded tissue array was incubated with an anti-IL-6 antibody (1:100 dilution) or an anti-IFIT2 antibody (1:400 dilution) for 15 min at room temperature. 3,3'-diaminobenzidine (DAB) staining was performed for 10 min, and hematoxylin staining was performed for 10 min. IHC staining was analyzed by a well-trained pathologist and scored on a scale of 1 to 3, with 1 indicating weak expression, 2 indicating moderate expression, and 3 indicating high expression.

### Statistical analysis

The data are expressed as means ± Standard Deviation (SD). SigmaPlot version 13.0 (Systat Software, San Jose, CA) was used to create the figures. Student’s t-test was used to determine significant differences between two different groups. If the normality test failed, the comparison of two groups was performed using the Mann‒Whitney rank sum test. In in vitro experiments, differences between two groups were analyzed using Student’s t-test, whereas differences among multiple groups were assessed by one-way ANOVA with Fisher’s least significant difference (LSD) test as post-hoc analysis. A p value less than 0.05 was considered significant. The correlation between IL-6 and other cytokines was analyzed using the Spearman’s rank correlation coefficient test.

## Results

### Secreted IL-6 levels in IFIT2-depleted oral squamous cell carcinoma (OSCC) cells

**Fig. 1:**
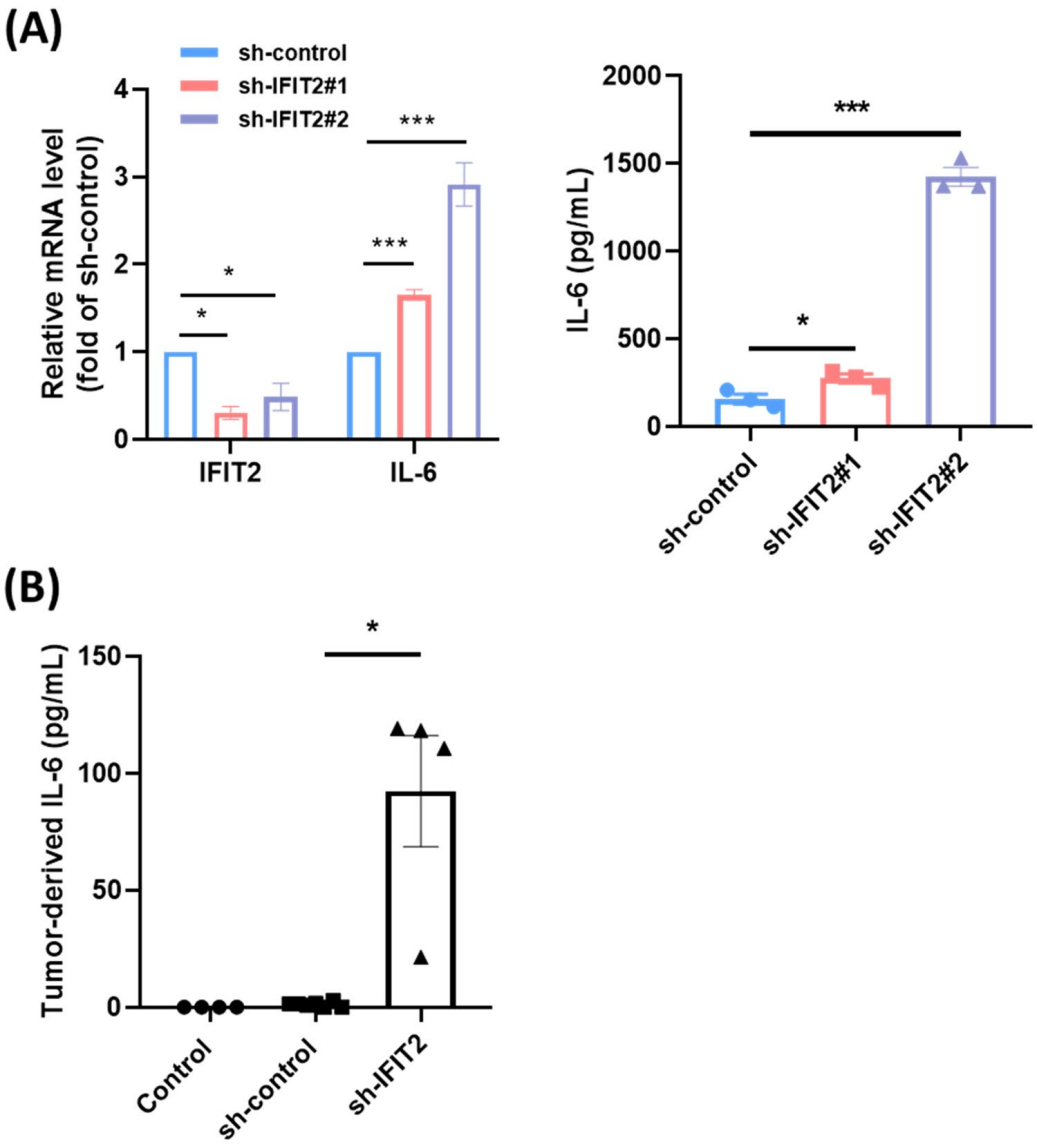
*Effect of IFIT2 depletion on IL-6 expression in OSCC cells.* (A) Q-PCR showing relative mRNA levels of IFIT2 and IL-6 in OSCC cells transduced with sh-control, sh-IFIT2#1, and sh-IFIT2#2 (left). IL-6 levels in CM of sh-IFIT2-depleted cells compared to sh-control cells (right). (B) Serum IL-6 levels in mice bearing sh-control, sh-IFIT2 tumors. Mice without tumor inoculation were used as the control group. Data are presented as mean ± SEM, with statistical significance indicated as *p < 0.05 and ***p < 0.001 compared to sh-control groups

It has been reported that IFIT2 depletion is involved in inflammatory processes. To comprehensively investigate IFIT2’s role in inflammation, we examined cytokine levels in conditioned media from IFIT2 knockdown OSCC cells. As shown in Table S1, knockdown of IFIT2 (sh-IFIT2#1 and sh-IFIT2#2) led to increased production of pro-inflammatory cytokines (IL-6, IL-8, TNFα), chemokines (MCP-1), and angiogenic factors (VEGF-A, PDGF-AA), compared to the control group (shCTRL). Notably, IL-6 and TNFα were significantly elevated. In vitro experiments further demonstrated that IFIT2 depletion (sh-IFIT2#1 and sh-IFIT2#2) markedly enhanced IL-6 secretion compared to the sh-control group (Fig. 1A). These findings suggest that IFIT2 loss directly promotes IL-6 production in OSCC cells. Additionally, tumor xenografts derived from IFIT2-depleted cells showed significantly higher IL-6 levels than control xenografts (Fig. 1B), aligning with the in vitro data. Other cytokines, including IL-10 and IL-4, displayed minimal changes, indicating a more selective effect of IFIT2 depletion on IL-6 and TNFα. This underscores IFIT2’s potential regulatory role in inflammation and tumor progression.

### IFIT2 depletion in OSCC promotes tumor-driven bone resorption via enhanced osteoclast differentiation

**Fig. 2:**
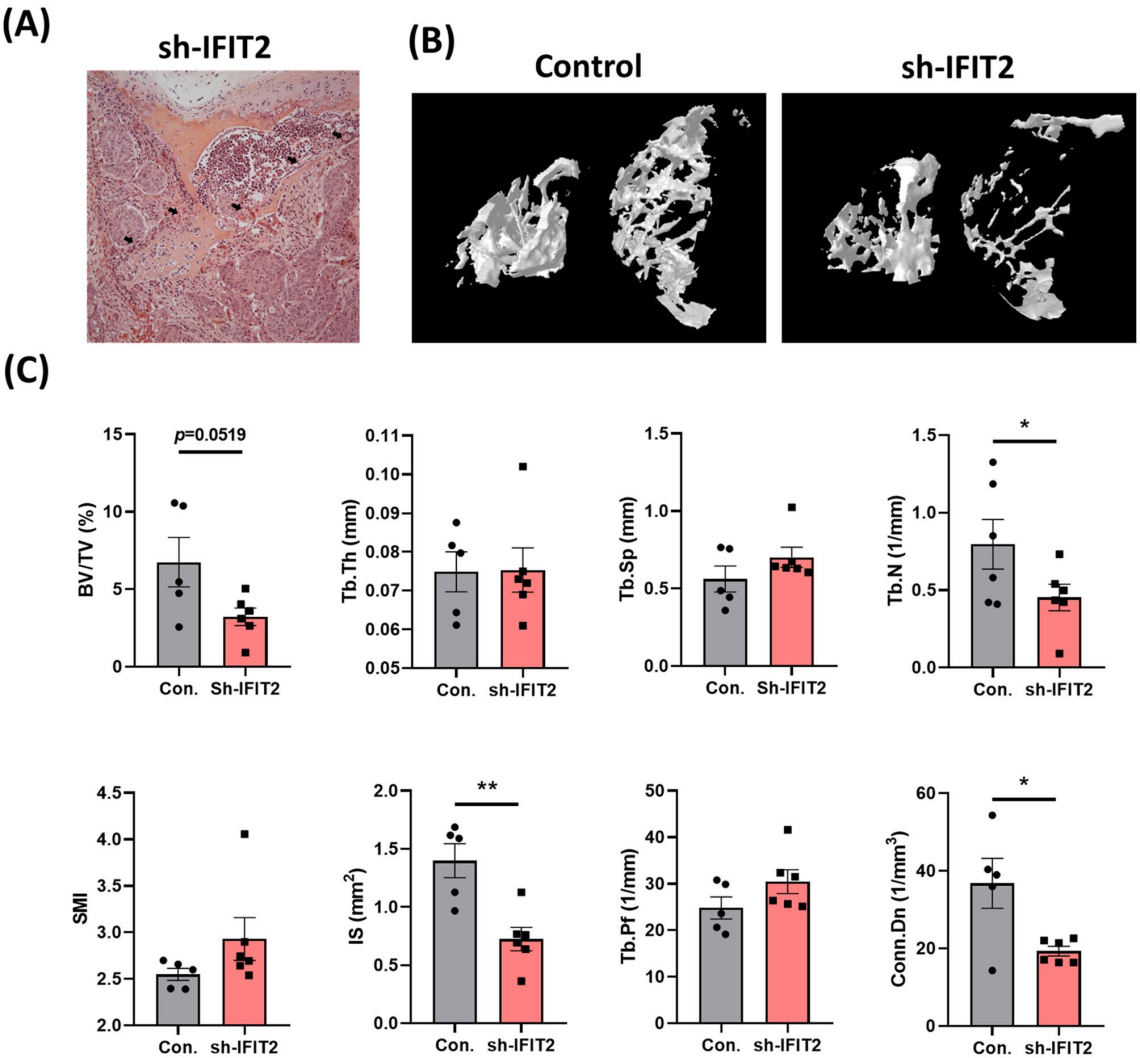
*IFIT2 depletion promotes tumor-associated bone invasion.* (A) Representative H&E stained sections showing obvious bone invasion by IFIT2-depleted tumor cells (arrows). (B) Representative 3D μCT reconstructions illustrating the trabecular microarchitecture in control vs. IFIT2-depleted tumor-bearing mice. (C) Quantitative μCT analysis of BV/TV, Tb.Th, and Tb.N in IFIT2-depleted mice compared with controls. Data are presented as mean ± SEM, with statistical significance indicated as *p < 0.05 and ***p < 0.001 compared to sh-control groups

**Fig. 3:**
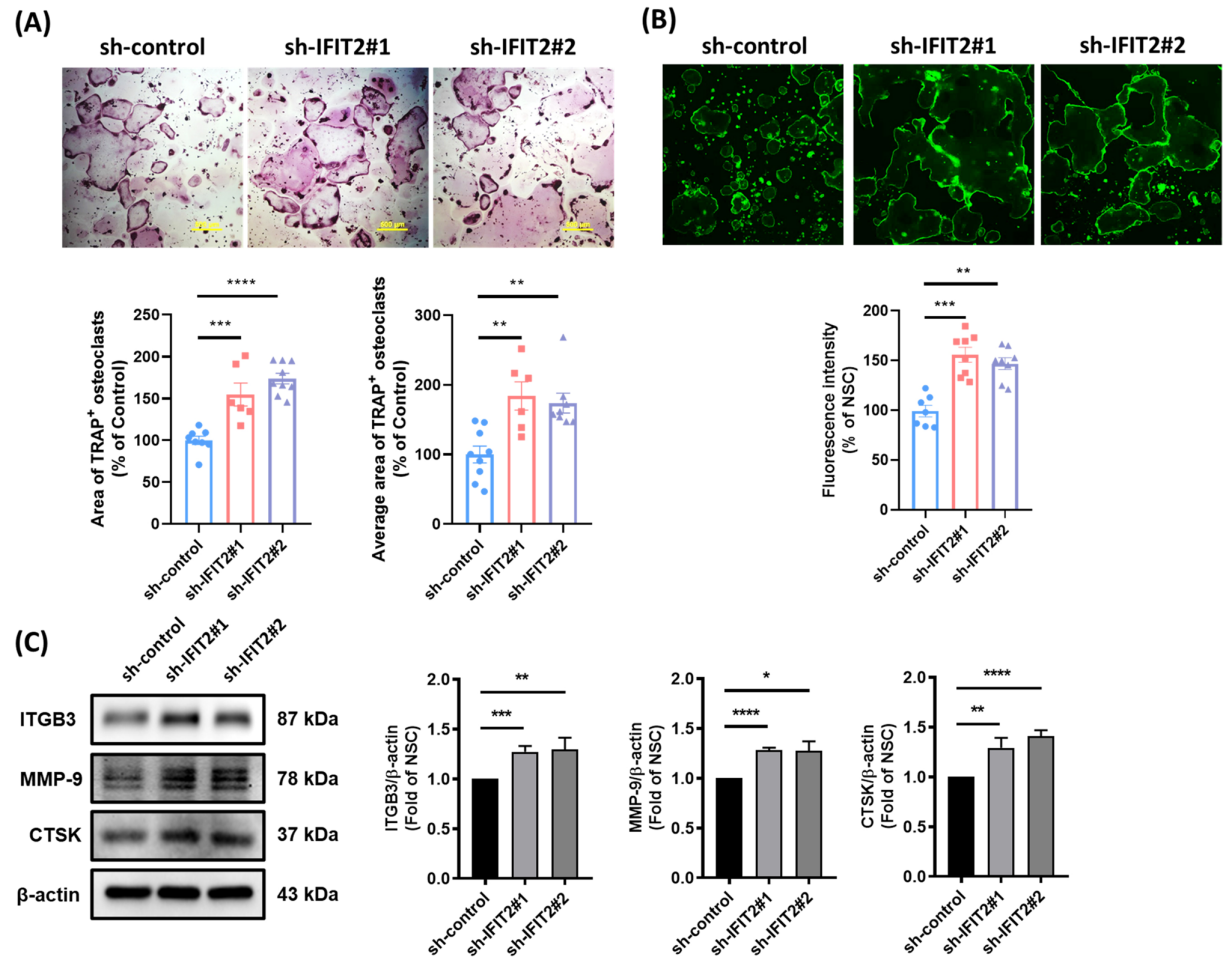
*IFIT2 depletion in OSCC cells enhances osteoclast formation and maturation.* RAW 264.7 precursor cells were cultured for 4 days with CM from sh-control or sh‐IFIT2 OSCC cells. **A** Representative images of TRAP-stained osteoclasts (upper panel). The lower panel shows the quantification of the total area and average area of TRAP-positive multinucleated cells. **B** Microscope images showing mature osteoclast actin ring structures in RAW 264.7 cells under the same culture conditions (upper panel). The lower panel presents the quantification of actin ring fluorescence intensity. **C** Total protein lysates were collected from osteoclasts. Western blot images showing the expression of osteoclast markers (ITGB3, MMP-9, and CTSK) (left panel). Quantification analysis of Western blots is shown in the right panel. Data are presented as mean ± SEM, with statistical significance indicated as *p < 0.05, **p < 0.01, ***p < 0.001, and ****p < 0.0001 compared to sh-control groups

Our previous research has shown that IFIT2 depletion enhances tumor colonization in multiple organs, including bone, leading to prominent bone invasion (Fig. 2A) [18]. To investigate this further, we performed microcomputed tomography (μCT) to assess bone microarchitecture. Quantitative μCT analysis indicated a reduction in bone volume fraction (BV/TV), trabecular thickness (Tb.Th), and trabecular number (Tb.N) in mice bearing IFIT2-depleted tumors (Fig. 2B and C), suggesting that IFIT2 loss contributes to tumor-mediated bone resorption. OSCC cells produce factors such as IL-6, IL-1β, IL-8, and PTHrP, which are known to promote osteoclast formation and bone invasion [28, 29]. To determine whether IFIT2 depletion augments osteoclast differentiation, we incubated RAW 264.7 osteoclast precursor cells with conditioned media (CM) from IFIT2-depleted OSCC cells. After four days, cells treated with sh-IFIT2 CM exhibited a marked increase in osteoclast formation compared to the sh-control group (Fig. 3A). Actin ring formation, a hallmark of mature osteoclasts, was also significantly elevated in the sh-IFIT2 CM groups (Fig. 3B). Moreover, key osteoclast differentiation markers—ITGB3, MMP-9, and CTSK—were upregulated in these groups (Fig. 3C). These data indicate that IFIT2 depletion in OSCC cells fosters bone resorption by enhancing osteoclast differentiation and activation.

### Elevated IL-6 expression in IFIT2-depleted OSCC cells promotes osteoclast formation and bone invasion

**Fig. 4:**
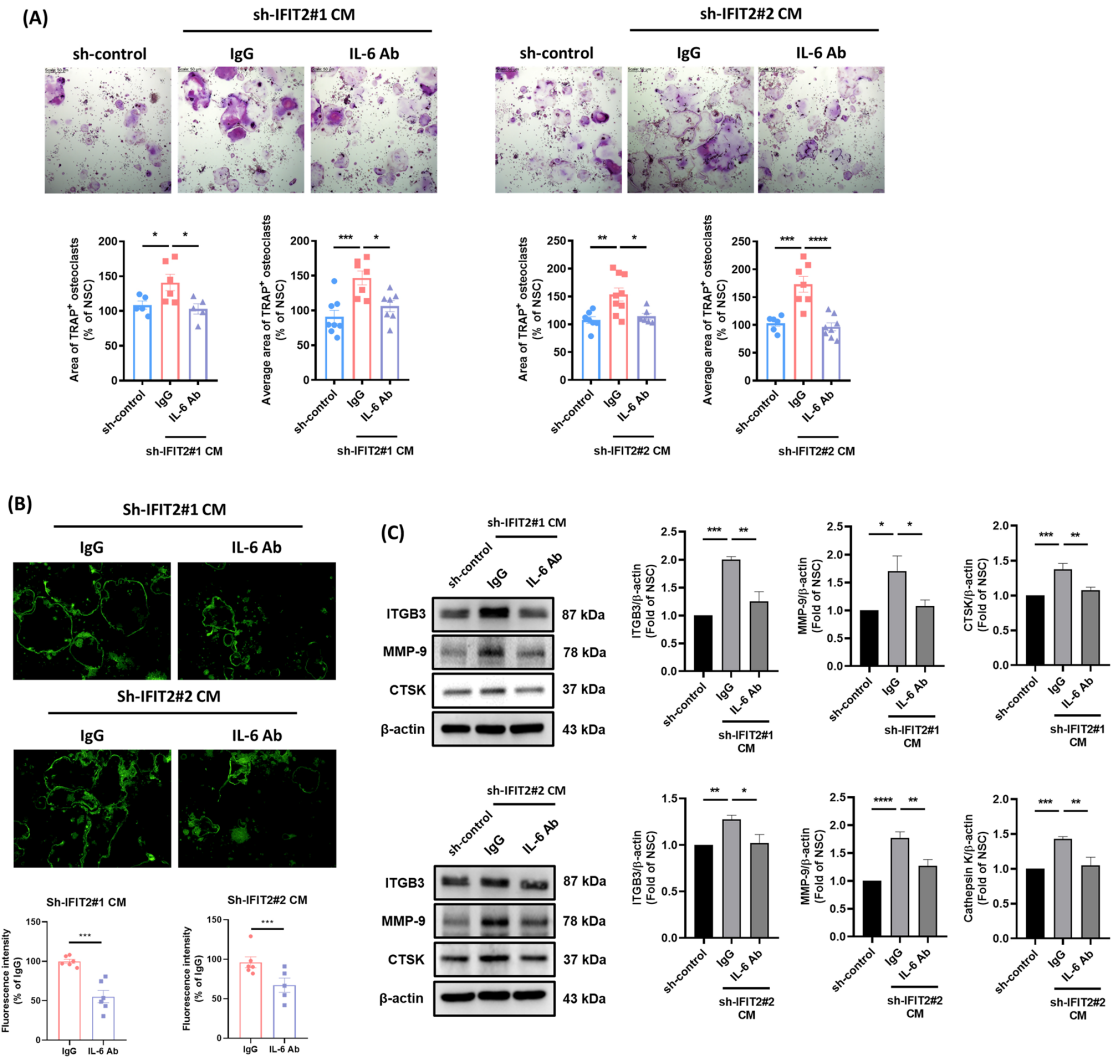
*IL-6 blockade reduces osteoclastogenesis induced by IFIT2 depletion in OSCC cells.* RAW 264.7 cells were cultured for 4 days with CM from sh-control or sh-IFIT2 OSCC cells, in the presence of an IL-6 neutralizing antibody or IgG control. **A** TRAP-stained images of osteoclasts (upper panel), with the lower panel showing the quantification of TRAP-positive multinucleated cells. **B** Microscope images of mature osteoclast actin ring structures (upper panel), with the lower panel presenting the quantification of actin ring fluorescence intensity. **C** Western blot images displaying the expression of osteoclast markers (ITGB3, MMP-9, and CTSK) in protein lysates collected from these osteoclasts (left panel), with quantification analysis shown in the right panel. Data are presented as mean ± SEM, with statistical significance indicated as *p < 0.05, **p < 0.01, ***p < 0.001, and ****p < 0.0001 compared to the IgG control group

IL-6 has been identified as a critical factor in osteoclast differentiation and tumor-induced bone destruction [30]. Consistent with these reports, our findings revealed that IL-6 was significantly upregulated in IFIT2-depleted OSCC cells. To evaluate the functional impact of this IL-6 elevation, we performed osteoclastogenesis assays using CM from IFIT2-depleted cells, either with or without an IL-6 neutralizing antibody. Blocking IL-6 significantly reduced sh-IFIT2 CM-induced osteoclast formation, actin ring formation, and expression of major osteoclast differentiation markers (Fig. 4A–C). These results confirm that IL-6 is a key mediator of bone destruction in IFIT2-depleted OSCC cells.

### Serum IL-6 levels in clinical OSCC patients

To comprehensively understand the clinical significance of IL-6 and other cytokines, serum samples from 47 OSCC patients were analyzed using cytokine profiling. As presented in Table 1, significant differences were observed for IL-6, G-CSF, and MDC between early and late clinical stages. IL-6 and G-CSF levels were significantly elevated in stage IV patients, while MDC levels were notably reduced. Specifically, G-CSF levels were higher in stage IV patients compared to stages I–III (38.21 vs. 27.0 pg/mL; *p* = 0.015), whereas MDC levels were significantly lower in stage IV patients (248.64 vs. 340.39 pg/mL; *p* = 0.046). Table 2 indicates that serum IL-6 levels were not significantly associated with sex or age. However, significant elevations in serum IL-6 levels were identified in patients with advanced tumor stages (T4 vs. T1–3; 22.21 vs. 11.04, *p* = 0.027), lymph node involvement (N1–3 vs. N0; 24.38 vs. 14.4, *p* = 0.027), and stage IV compared to stages I–III (21.31 vs. 11.68, *p* = 0.043). No significant differences were observed regarding tumor differentiation status. These findings suggest that elevated serum IL-6 levels correlate with advanced tumor stages and lymph node metastasis in OSCC. Furthermore, correlation analyses (Table 3) revealed significant associations between IL-6 and several cytokines, underscoring IL-6’s central role in the inflammatory and tumor microenvironment. G-CSF exhibited the strongest positive correlation with IL-6 (r = 0.706, *p* < 0.0001), followed by IFN-α2 (r = 0.543, *p* < 0.01), IL-9 (r = 0.454, *p* < 0.01), and VEGF-α (r = 0.454, *p* < 0.01). IL-1α had a weaker yet significant correlation (r = 0.388, *p* < 0.05). Conversely, FLT-3L showed a moderate negative correlation (r = -0.436, *p* < 0.05). These findings suggest IL-6 is tightly linked with pro-inflammatory and growth-promoting cytokines, emphasizing its potential role in cancer progression and the tumor microenvironment.

**Table 1:** Serum cytokine changes in 47 patients with OSCC

	Stage 1–3 Mean (SEM)	Stage 4 Mean (SEM)	*p* value ^**#**^
sCD40L	2436.865 (654.801)	3294.193 (593.438)	0.515
EGF	101.617 (28.745)	94.402 (32.445)	0.246
Eotaxin	61.875 (11.016)	49.476 (7.183)	0.231
FGF-2	51.569 (9.634)	55.451 (15.693)	0.320
FLT-3L	8.377 (1.038)	6.958 (0.594)	0.299
Fractalkine	125.091 (21.911)	154.772 (19.919)	0.386
G-CSF	26.998 (9.908)	38.211 (4.774)	**0.015**
GRO-α	16.415 (4.369)	26.394 (8.966)	0.310
IFN-α2	6.398 (1.655)	10.281 (2.379)	0.412
IFN-γ	6.231 (4.134)	5.481 (2.456)	0.720
IL-1α	0.884 (0.152)	1.748 (0.859)	0.806
IL-1β	10.21 (5.211)	17.167 (7.767)	0.675
IL-1RA	2.572 (0.301)	4.004 (0.938)	0.802
IL-2	1.066 (0.268)	25.065 (24.821)	0.571
IL-4	2.851 (1.585)	1.26 (0.169)	0.862
IL-5	2.173 (0.358)	1.708 (0.231)	0.355
IL-6	11.044 (2.19)	22.205 (3.607)	**0.027**
IL-8	11.625 (1.468)	11.922 (1.14)	0.927
IL-9	11.048 (3.086)	13.164 (2.005)	0.451
IL-10	21.687 (6.971)	20.443 (4.146)	0.501
IL-12 (p40)	28.3 (5.433)	28.602 (6.435)	0.706
IL-12 (p70)	2.688 (0.493)	8.121 (4.786)	0.799
IL-13	19.693 (4.3)	24.561 (3.791)	0.570
IL-15	8.101 (0.779)	7.589 (0.522)	0.608
IL-17A	3.705 (0.645)	13.465 (9.804)	0.689
IL-17E/IL-25	47.367 (6.816)	38.948 (5.982)	0.123
IL-17F	9.77 (1.742)	19.092 (9.539)	0.584
IL-18	8.024 (1.145)	7.407 (0.665)	0.724
IL-22	28.357 (13.926)	72.244 (31.435)	0.216
IL-27	499.783 (100.342)	534.766 (73.499)	0.545
IP-10	93.158 (22.537)	85.979 (12.514)	0.925
MCP-1	725.62 (176.827)	459.48 (38.427)	0.259
MCP-3	14.303 (1.082)	14.779 (1.303)	0.648
M-CSF	63.427 (13.007)	78.323 (10.496)	0.405
MDC	340.393 (42.055)	248.64 (18.08)	**0.046**
MIG	1058.426 (140.131)	988.932 (118.387)	0.545
MIP-1α	– (–)	110.87 (–)	
MIP-1β	25.625 (4.628)	20.326 (1.639)	0.767
PDGF-AA	4095.306 (421.063)	4104.883 (332.167)	0.656
PDGF-AB/BB	6290.854 (595.178)	6778.914 (329.332)	0.442
RANTES	2013.804 (169.226)	2201.179 (195.394)	0.991
TGF-α	3.856 (0.523)	6.765 (2.629)	0.621
TNF-α	10.949 (1.369)	11.947 (1.105)	0.616
TNF-β	2.638 (0.991)	12.308 (4.02)	0.222
VEGF-α	157.04 (37.28)	176.826 (22.856)	0.278

^#^By t-test

**Table 2:** Association of serum IL-6 levels with clinical variants in 47 OSCC patients

Variable	No. of cases	IL-6 (SEM)	*p* value ^#^
*Sex*			
Male	44	46.66 (30.02)	0.361
Female	3	16.73 (1.96)	
Age < 60	26	16.54 (2.78)	0.277
≥ 60	21	21.48 (5.0)	
*Tumor stage*			
T1-3	15	11.04 (2.19)	**0.027**
T4	32	22.21 (3.61)	
*Lymph node stage*			
N0	27	14.4 (2.57)	**0.027**
N1-3	20	24.38 (5.0)	
*Clinical stage*			
I–III	13	11.68 (2.9)	**0.043**
IV	34	21.31 (3.4)	
*Differentiation*			
Moderate + poor	32	18.02 (4.29)	0.891
Well	15	18.93 (3.38)	

^#^By t-test

**Table 3:** Summary of correlations between IL-6 and cytokines in stage 4 OSCC patients

Cytokine	Correlation Coefficient (r)	*p* value
G-CSF	0.706	2E-07
IFN-α2	0.543	0.00207
IL-9	0.454	0.00921
VEGF-α	0.454	0.00928
IL-1α	0.388	0.0452
FLT-3L	−0.436	0.0128

### Correlation between IL-6/IFIT2 ratio and survival outcomes

**Fig. 5:**
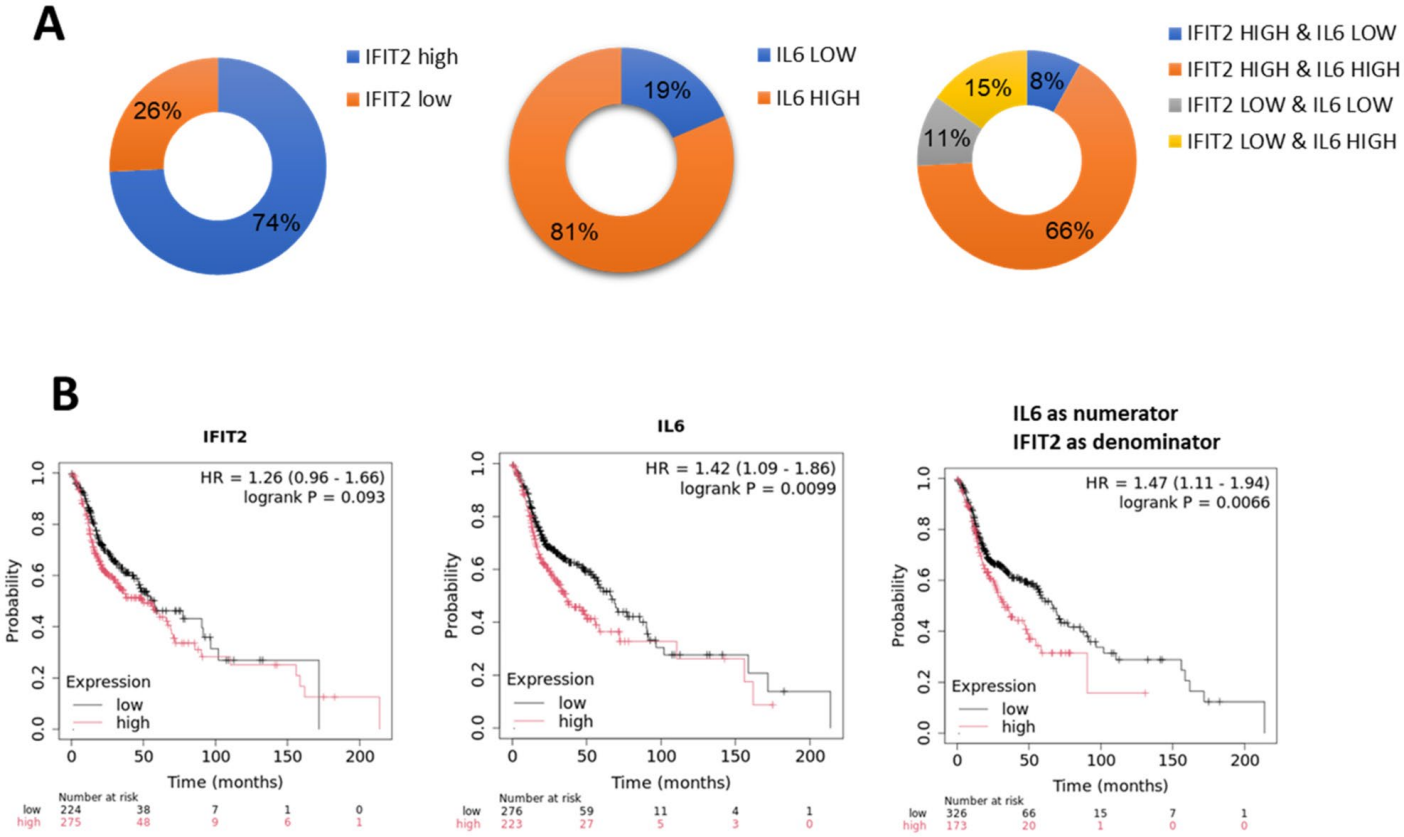
*Analysis of IFIT2 and IL-6 expression in HNC and their prognostic significance.* **A** Pie charts showing the distribution of IFIT2 and IL-6 expression levels in HNC samples. **B** Kaplan–Meier survival curves illustrating the prognostic impact of IFIT2, IL-6, and the IL-6/IFIT2 expression ratio in HNC patients

The oral cancer tissue array was stratified based on IL-6 and IFIT2 expression levels to investigate their relationship. As shown in Fig. 5A, a majority (77%) of patients demonstrated a positive correlation between IL-6 and IFIT2 expression, indicating that high IFIT2 levels were associated with high IL-6 levels, and low IFIT2 levels were linked with low IL-6 levels. Conversely, the remaining 23% of patients exhibited a negative correlation, where low IFIT2 expression corresponded with high IL-6 levels or high IFIT2 expression corresponded with low IL-6 levels. This data highlights a predominant association between IL-6 and IFIT2 expression in OSCC but also underscores a subset of tumors with distinct regulatory patterns, suggesting possible tumor heterogeneity or alternative pathways driving IL-6 production. Clinical analysis of OSCC patients revealed that a high IL-6/IFIT2 ratio was associated with significantly reduced median survival (Fig. 5B, 32.93 months vs. 66.73 months for the low ratio group; *p* < 0.01, Mann–Whitney Rank Sum Test). This suggests that the IL-6/IFIT2 axis may be more relevant in the context of advanced disease.

### Correlation between IL-6/IFIT2 ratio and survival outcomes

To examine the relationship between IL-6 and IFIT2 expression in OSCC, oral cancer tissue arrays were stratified accordingly. As illustrated in Fig. 5A, most patients (77%) exhibited a positive correlation, where high IL-6 expression aligned with high IFIT2 expression, and low IL-6 with low IFIT2 levels. In contrast, the remaining 23% displayed an inverse relationship, where low IFIT2 expression corresponded with high IL-6 levels or vice versa. This indicates predominant but heterogeneous regulatory patterns between IL-6 and IFIT2. Clinical analysis (Fig. 5B) demonstrated that patients with a high IL-6/IFIT2 ratio experienced significantly reduced median survival (32.93 vs. 66.73 months; *p* < 0.01, Mann–Whitney Rank Sum Test), suggesting the IL-6/IFIT2 axis as an important prognostic factor in advanced OSCC.

## Discussion

This study identifies IL-6 as a critical mediator of bone invasion in IFIT2-depleted OSCC, providing both mechanistic insights and clinical implications. Elevated IL-6 levels in the context of reduced IFIT2 expression were observed consistently in both in vitro and in vivo models, suggesting a direct regulatory role. These findings are further supported by clinical data showing that a high IL-6/IFIT2 ratio predicts poor survival outcomes, particularly in advanced-stage head and neck cancer, including OSCC.

IL-6 is widely recognized as a key mediator of bone invasion and metastasis in various malignancies, primarily through its capacity to enhance osteoclast differentiation, promote inflammatory signaling, and remodel the bone microenvironment [30–33]. IL-6 primarily stimulates osteoclastogenesis and bone resorption [34]. Its effects are closely linked with IL-1, TNF, and PTHrp, as IL-6 promotes IL-1 release, which subsequently enhances osteoclast formation [35]. Previous study found that IL-6R neutralizing antibody directly inhibited osteoclast formation both in vitro and in vivo, indicating that anti-IL-6R therapy specifically targets osteoclasts, independent of its anti-inflammatory properties [36]. Consistent with these observations, our current findings reveal that depleting IFIT2 significantly induces IL-6 expression in OSCC cells. This upregulation of IL-6 may facilitate osteoclast-mediated bone resorption and tumor expansion within bone tissue. Elevated IL-6 levels in OSCC have been associated with enhanced cell proliferation, angiogenesis, and immune evasion. Furthermore, IL-6 activates downstream signaling pathways, such as the JAK/STAT3 and MAPK pathways, which contribute to the invasive and metastatic capabilities of OSCC cells [37]. Moreover, high IL-6 levels are associated with poor prognosis in OSCC patients [38]. These findings position IL-6 not only as a biomarker for disease severity but also as a potential therapeutic target to limit tumor progression and bone invasion in OSCC. In recent years, targeting IL-6 or its receptor (IL-6R) has gained increasing attention as a promising strategy to mitigate tumor-induced bone metastasis and bone loss [39]. Clinically, monoclonal antibodies that block IL-6 signaling—such as tocilizumab, sarilumab, and siltuximab—have been used for autoimmune diseases like rheumatoid arthritis [40–43], demonstrating both acceptable safety profiles and significant suppression of bone degradation. Given the critical role of the bone microenvironment in tumor growth, invasion, and metastasis, further evaluation of these IL-6 inhibitors in OSCC bone invasion or metastasis may aid in developing new, more precise therapeutic approaches.

The marked increase in IL-6, IL-8, and TNFα upon IFIT2 knockdown suggests IFIT2’s potential role in suppressing inflammatory pathways [44]. This aligns with findings that IFIT2 may act as a tumor suppressor, and its downregulation exacerbates pro-tumor inflammation. A previous study highlights the role of IFIT2 in inflammation by demonstrating that overexpressing IFIT2 in RAW264.7 macrophage cells specifically suppresses the LPS-induced production of pro-inflammatory cytokines such as TNF-α and IL-6 [21]. In osteoporosis, pro-inflammatory cytokines such as TNF-α, IL-1β, RANKL, IL-6, and IL-17 are markedly elevated, while IL-10, a key inhibitor of osteoclastogenesis, is downregulated, thereby facilitating osteoclast development [45]. In our study, we observed that silencing IFIT2, a known suppressor of these pro-inflammatory pathways, significantly enhances osteoclast formation and promotes bone invasion in OSCC. These findings underscore the pivotal role of IFIT2 as a regulator of inflammation-mediated osteoclastogenesis and highlight its potential as a therapeutic target for mitigating bone invasion in OSCC. Furthermore, previous study finds the suppression of TNF-α by IFIT2 is caused by the destabilization of TNF-α mRNA [21], potentially through binding AU-rich elements in their 3’ untranslated regions [46]. As IL-6 is a primary mediator of inflammatory responses, controlling its production via post-transcriptional regulation could prove particularly critical. Nonetheless, the precise mechanism by which IFIT2 regulates IL-6 expression still remains largely unclear and requires further verification.

High-throughput screening of IFIT2 depletion in OSCC cells has been previously reported, and we found that IFIT2 may play an important role in inflammation [20, 47]. In the present study, knockdown of IFIT2 (sh-IFIT2#1 and sh-IFIT2#2) in OSCC cells led to increased production of the pro-inflammatory cytokines IL-6 and TNF-α by cytokine profiling. Blocking TNF-α inhibits cancer stem cell-like phenotypes, in vivo angiogenesis and the growth of IFIT2‑depleted OSCC cells [20, 48]. However, we found that treatment with a TNF-α-neutralizing antibody did not reduce sh-IFIT2 CM-induced osteoclast formation (data not shown). Levels of granulocyte–macrophage colony-stimulating factor (GM-CSF), growth related oncogene-α (GRO-α), IL-6, IL-8, IL-18, interferon gamma-induced protein 10 (IP-10), CCL2, CCL22, and TNF-α were elevated in cachectic mice bearing metastatic IFIT2-depleted tumors [23]. IL-6 stimulates the expression of several pro-inflammatory and pro-angiogenic mediators, including IL-8, CCL2, GM-CSF, and VEGF, which act in an autocrine and/or paracrine fashion to influence both immune and non-immune cells within the tumor microenvironment [49]. Future studies will continue to investigate IL-6 and its associated inflammatory cytokines involved in IFIT2-depletion-induced bone invasion.

Analyzing serum cytokine levels may aid in identifying patients with a poor prognosis who could benefit from more intensive disease management [50]. The present study reveals significant correlations between IL-6 and several cytokines, including G-CSF, IFN-α2, IL-9, and VEGF-α. Biomarker potential of IL-6 and VEGF-α was evaluated in ascitic fluid of epithelial ovarian cancer patients [51]. IL-6-induced VEGF secretion was significantly suppressed by a PI3K inhibitor (LY294002), and it was accompanied by inhibited phosphorylation of Akt in prostate cancer cells [52] . IL-1a demonstrated a weaker but significant positive correlation. Conversely, FLT-3L exhibited a moderate negative correlation with IL-6. These results suggest IL-6 is tightly linked with pro-inflammatory and growth-promoting cytokines, emphasizing its potential role in cancer progression and the tumor microenvironment. Notably, G-CSF displays the strongest positive correlation with IL-6, while FLT-3L is negatively correlated. It has been reported that IL-6 cooperates with G-CSF to induce pro-tumor function of neutrophils in bone marrow by enhancing STAT3 activation [53].The observed negative correlation of IL-6 with FLT-3L is intriguing, as FLT-3L is associated with dendritic cell differentiation and antitumor immunity [54]. This could suggest immune evasion mechanisms in advanced OSCC. The findings suggest that IL-6 levels may be tightly linked with the pro-inflammatory and growth-promoting cytokine milieu, potentially playing a critical role in the tumor microenvironment or inflammatory processes associated with cancer progression. Additional in vivo studies in mouse models are warranted to further confirm the inhibitory effects of IL-6 neutralizing antibody on the progression of IFIT2-depleted OSCC cells. Moreover, further research is essential to validate these associations and elucidate their mechanistic basis.

## Conclusion

This study demonstrates the importance of cytokine profiles in OSCC progression and highlights IFIT2 as a critical regulator of inflammatory responses. This work underscores the prognostic and therapeutic relevance of the IL-6/IFIT2 axis in OSCC, paving the way for future investigations into targeted interventions in high-risk patient subgroups. Targeting IL-6 and related cytokine pathways could provide therapeutic benefits in managing OSCC, particularly in advanced stages.

## Supplementary Information

Below is the link to the electronic supplementary material.

Supplementary file1 (DOCX 20 KB)

Supplementary file2 (PDF 603 KB)

## Data Availability

Data will be made available on request.
