# Supplementary material for: IL-6 as a driver of bone invasion in IFIT2-depleted oral squamous cell carcinoma
Source: Cancer Immunol Immunother. 2026 Jan 31;75(2):64. doi: 10.1007/s00262-025-04234-6 (PMC12860772; doi:10.1007/s00262-025-04234-6)
Supplement: Supplementary file 1 — Supplementary file1 (DOCX 20 KB) [file 262_2025_4234_MOESM1_ESM.docx]

**Table S1. Summary of cytokines in IFIT2 depleted OSCC cells**

| Cytokines | Sh-control  Mean (SEM) | shIFIT2-1  Mean (SEM) | shIFIT2-2  Mean (SEM) |
| --- | --- | --- | --- |
| sCD40L | 185.58 (105.49) | 339.43 (11.39) | 306.43 (104.48) |
| EGF | 12.03 (3.4) | 14.53 (1.52) | 11.58 (2.99) |
| Eotaxin | 15.65 (5.51) | 21.25 (1.01) | 22.09 (5.13) |
| FGF-2 | 84.79 (23.03) | 188.55 (37.49) | 276.94 (34.66) |
| FLT-3L | 4.28 (2.32) | 3.83 (0.64) | 5.15 (1.45) |
| Fractalkine | 281.95 (111.22) | 278.29 (6.14) | 317.66 (65.48) |
| G-CSF | 18.67 (10.15) | 30.55 (3.83) | 29.17 (8.17) |
| GM-CSF | 90.61 (37.34) | 119.1 (12.15) | 958.24 (212.13) |
| GRO-α | 1436.47 (451.18) | 741.54 (236.87) | 2707.49 (155.01) |
| IFN-α2 | 18.33 (11.67) | 48.96 (3.57) | 34.77 (12.87) |
| IFN-γ | 9.45 (4.05) | 7.68 (0.65) | 12.9 (2.12) |
| IL-1α | 43.1 (18.8) | 24.7 (3.44) | 92.19 (23.99) |
| IL-1β | 7.52 (5.21) | 14.1 (1.15) | 16.9 (7.01) |
| IL-1RA | 2.49 (0.68) | 1.41 (0.12) | 3.14 (1.11) |
| IL-2 | 1.09 (0.67) | 1.04 (0.3) | 2.45 (1.19) |
| IL-4 | 5.22 (3.47) | 7.54 (0.73) | 10.17 (3.22) |
| IL-5 | 0.62 (0.26) | 0.71 (0.09) | 0.93 (0.31) |
| IL-6 | 158.14 (28.41) | 275.12 (26.3) | 1426.09 (52.95) |
| IL-8 | 1673.2 (931.43) | 872.54 (204.68) | 4041.25 (302.55) |
| IL-9 | 19.09 (12.53) | 34.8 (2.23) | 33.51 (10.82) |
| IL-10 | 2.22 (0.94) | 2.28 (0.23) | 2.86 (0.91) |
| IL-12 (p40) | 17.2 (8.07) | 16.11 (2.44) | 17.84 (4.24) |
| IL-12 (p70) | 5.41 (2.84) | 5.7 (0.53) | 7.2 (2.61) |
| IL-13 | 41.08 (30.21) | 87.65 (1.81) | 85.16 (31.84) |
| IL-15 | 7.56 (4.38) | 10.85 (0.57) | 13.61 (4.93) |
| IL-17A | 8.1 (5.26) | 13.4 (1.25) | 12.65 (5.6) |
| IL-17E/IL-25 | 55.24 (37.67) | 80.93 (10.59) | 111.18 (45.84) |
| IL-17F | 6.74 (3.72) | 8.84 (0.75) | 12.45 (4.58) |
| IL-18 | 1.61 (0.98) | 1.92 (0.28) | 2.78 (0.97) |
| IL-22 | 80.95 (26.98) | 37.85 (2.94) | 68.32 (18.26) |
| IL-27 | 112.76 (32.19) | 141.7 (7.09) | 178.64 (27.67) |
| IP-10 | 1274.41 (781.43) | 343.72 (204.49) | 808.5 (84.57) |
| MCP-1 | 18283.77 (646.9) | 17113.57 (1543.38) | 17952.27 (673.89) |
| MCP-3 | 25.39 (11.28) | 41.41 (1.79) | 42.09 (10.25) |
| M-CSF | 752.95 (376.07) | 575.68 (21.08) | 1377.09 (309.76) |
| MDC | 20.31 (7.06) | 5.26 (0.25) | 12.23 (2.24) |
| MIG | 66.94 (12.68) | 21.91 (1.19) | 48.76 (5.22) |
| MIP-1β | 1.24 (0.41) | 0.74 (0.29) | 1.53 (0.51) |
| PDGF-AA | 1015.51 (390.32) | 1297.37 (62.45) | 864.57 (170.53) |
| PDGF-AB/BB | 503.5 (42.14) | 583.54 (14.93) | 622.7 (14.87) |
| RANTES | 325.4 (143.4) | 75.19 (25.55) | 281.82 (32.41) |
| TGF-α | 2.34 (0.89) | 2 (0.33) | 2.82 (0.77) |
| TNF-α | 33.06 (1.15) | 57.12 (2.66) | 161.58 (15.97) |
| TNF-β | 30.55 (18.64) | 42.27 (3.02) | 49.27 (12.07) |
| VEGF-α | 1185.16 (701.54) | 1648.2 (174.14) | 2582.15 (405.42) |
