# Supplementary figures and images for: IL-6 as a driver of bone invasion in IFIT2-depleted oral squamous cell carcinoma
Source: Cancer Immunol Immunother. 2026 Jan 31;75(2):64. doi: 10.1007/s00262-025-04234-6 (PMC12860772; doi:10.1007/s00262-025-04234-6)

sh-control  
sh-IFIT2#1  
sh-IFIT2#2

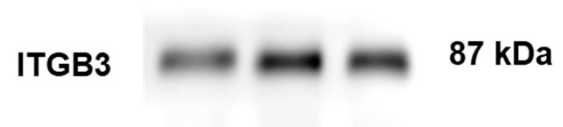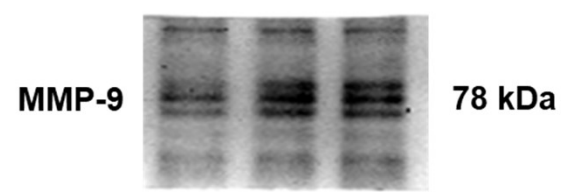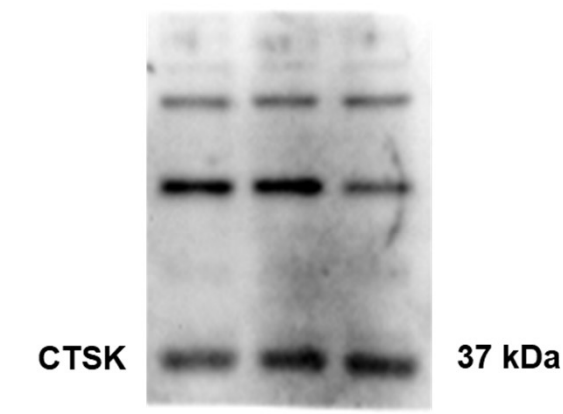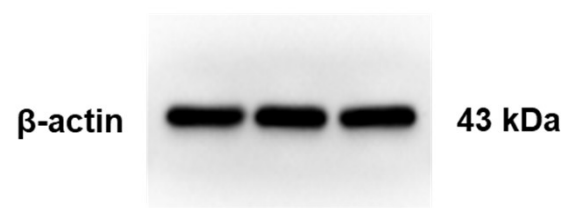

sh-control  
sh-IFIT2#1  
sh-IFIT2#2

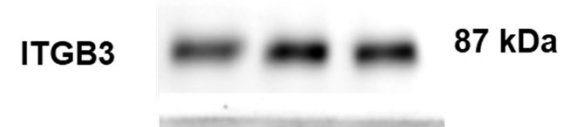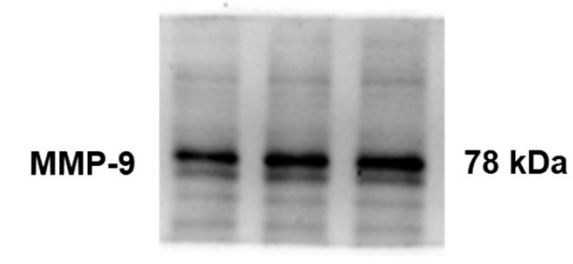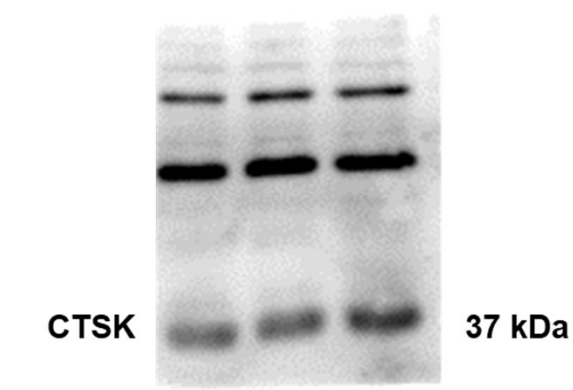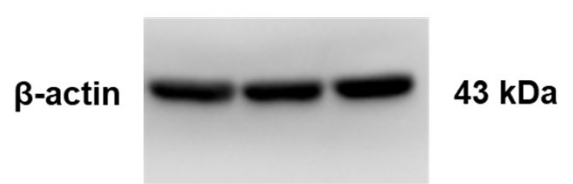

sh-control  
sh-IFIT2#1  
sh-IFIT2#2

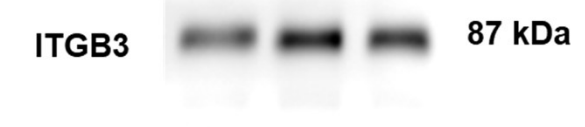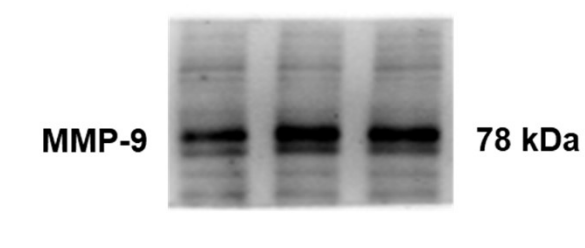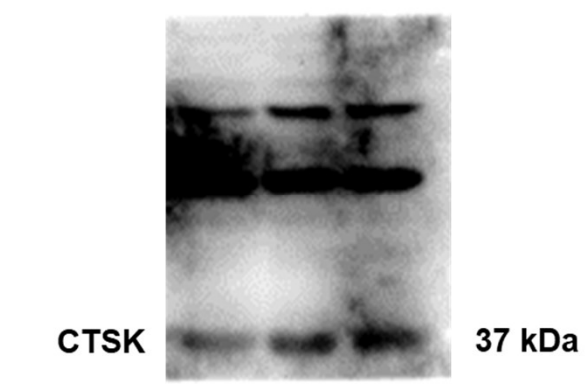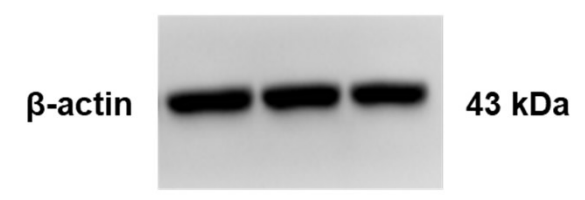

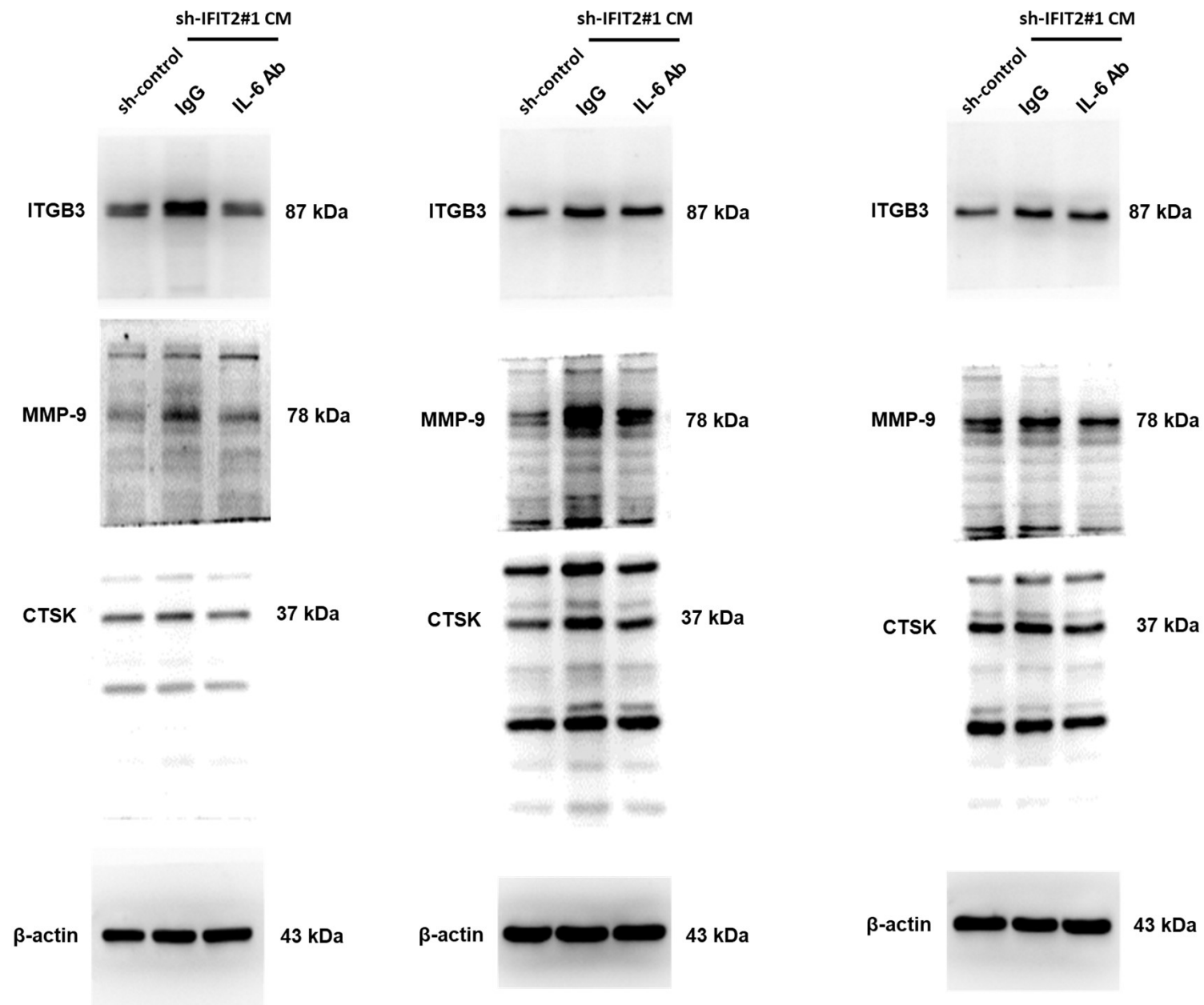

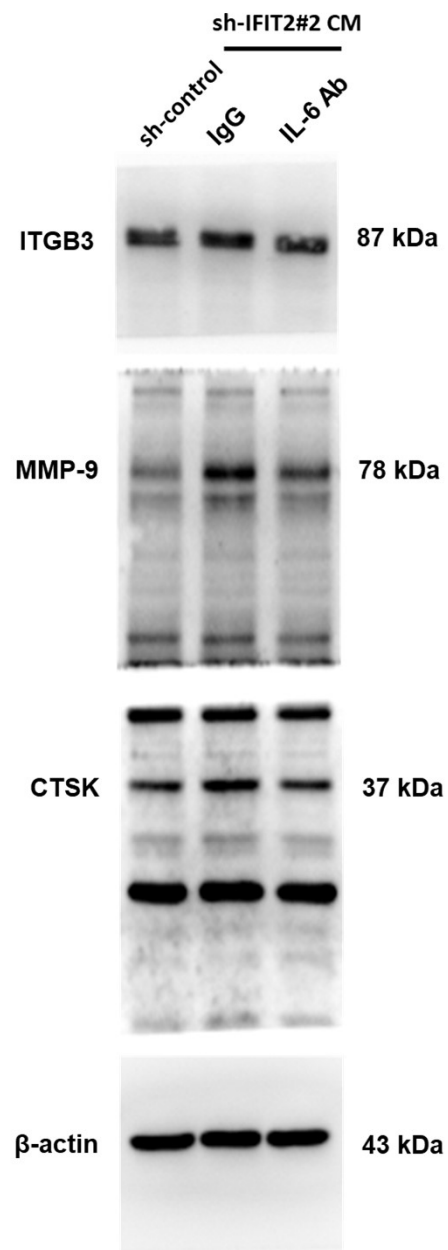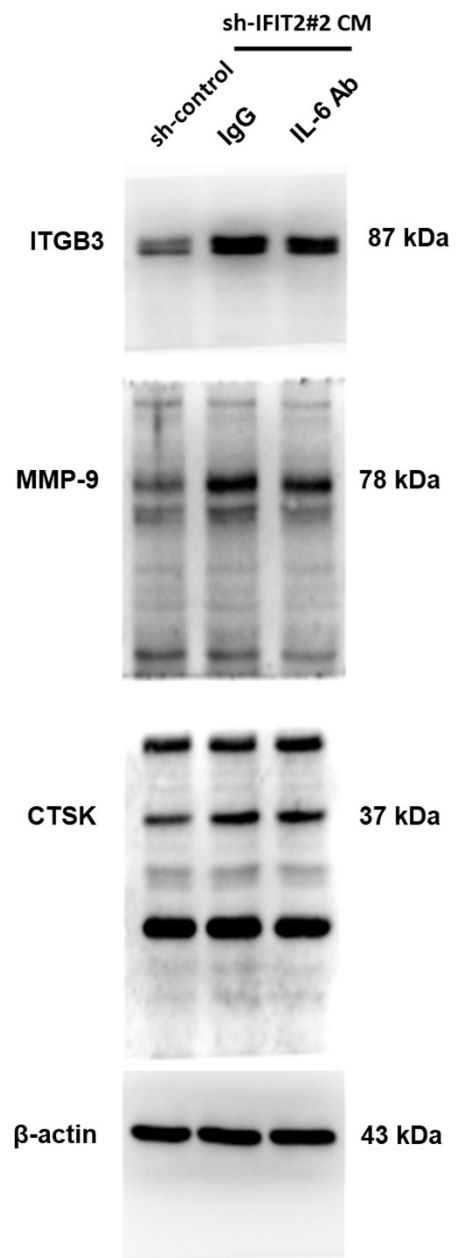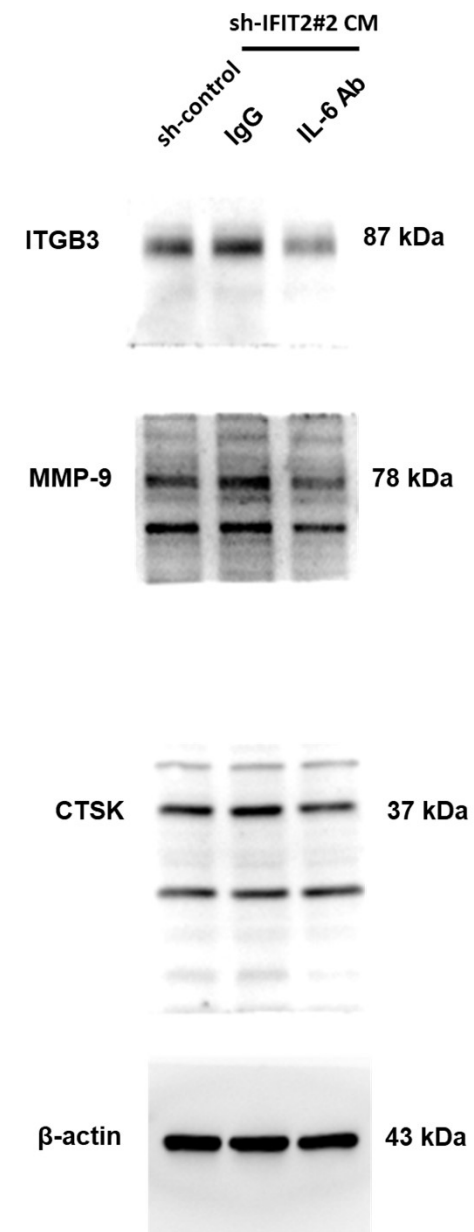

Supplement: Supplementary file 2 — Supplementary file2 (PDF 603 KB) [file 262_2025_4234_MOESM2_ESM.pdf]
